# Interpersonal Relationships in Assisted Living: Findings From a Survey of Family Members and Staff

**DOI:** 10.1093/geroni/igaa057.1235

**Published:** 2020-12-16

**Authors:** Francesca Falzarano, M Cary Reid, Leslie Schultz, Karl Pillemer

**Affiliations:** 1 Weill Cornell Medicine, New York, New York, United States; 2 Weill Cornell Medical College, New York City, New York, United States; 3 Cornell University, Ithaca, New York, United States

## Abstract

In recent decades, assisted living facilities (ALFs) have grown dramatically as an alternative to nursing homes. Research in nursing homes has shed light on the nature of the relationships that exist between family members and staff. However, little is known about family-staff relations within ALFs. We present data from the first study to describe the prevalence of conflict and positive and negative family-staff interactions in ALFs and to examine whether positive and negative aspects of the relationship contribute to salient staff and family outcomes. We use data collected from 252 family members and 472 staff members across 20 ALFs from the Partners in Care in Assisted Living (PICAL) study. Participants completed measures including interpersonal conflict, depression, perception of treatment, and stress-related to caregiving. Results showed that conflict among family and staff members is relatively low in ALFs. For staff, interpersonal conflict and treatment by family members significantly predicted burnout and depression. For families, only gender significantly predicted burden. Subgroup analyses, however, indicated that the effect of interpersonal conflict was significantly associated with perceived caregiver burden among family members whose relative has dementia. Despite the relatively harmonious relationships identified among family members and staff in ALFs, sources of conflict and negative interactions were identified, revealing the influence these relationships have on both family and staff outcomes. These findings can inform intervention efforts targeting family-staff interactions within ALFs.

